# Correction to: Evaluation of serological lateral flow assays for severe acute respiratory syndrome coronavirus-2

**DOI:** 10.1186/s12879-021-06333-y

**Published:** 2021-07-01

**Authors:** Bianca A. Trombetta, Savannah E. Kandigian, Robert R. Kitchen, Korneel Grauwet, Pia Kivisäkk Webb, Glenn A. Miller, Charles G. Jennings, Sejal Jain, Samara Miller, Yikai Kuo, Thadryan Sweeney, Tal Gilboa, Maia Norman, Daimon P. Simmons, Christopher E. Ramirez, Melissa Bedard, Catherine Fink, Jina Ko, Esmarline J. De León Peralta, Gerald Watts, Emma Gomez-Rivas, Vannessa Davis, Rocky M. Barilla, Jianing Wang, Pierre Cunin, Samuel Bates, Chevaun Morrison-Smith, Benjamin Nicholson, Edmond Wong, Leena El-Mufti, Michael Kann, Anna Bolling, Brooke Fortin, Hayden Ventresca, Wen Zhou, Santiago Pardo, Megan Kwock, Aditi Hazra, Leo Cheng, Q. Rushdy Ahmad, James A. Toombs, Rebecca Larson, Haley Pleskow, Nell Meosky Luo, Christina Samaha, Unnati M. Pandya, Pushpamali De Silva, Sally Zhou, Zakary Ganhadeiro, Sara Yohannes, Rakiesha Gay, Jacqueline Slavik, Shibani S. Mukerji, Petr Jarolim, David R. Walt, Becky C. Carlyle, Lauren L. Ritterhouse, Sara Suliman

**Affiliations:** 1 Department of Neurology, Massachusetts General Hospital, Harvard Medical School, Charlestown, Boston, MA USA; 2 Department of Medicine, Harvard Medical School, Boston, MA USA; 3 Mass General Brigham Innovation, Boston, MA USA; 4 Cardiology Division, Massachusetts General Hospital, Charlestown, MA USA; 5 Department of Neurosurgery, Brigham and Women’s Hospital, Harvard Medical School, Boston, MA USA; 6 Department of Medical Oncology and Center for Cancer-Genome Discovery, Dana-Farber Cancer Institute, Boston, MA USA; 7 Department of Pathology, Harvard Medical School, Boston, MA USA; 8 Center for Regenerative Medicine, Massachusetts General Hospital, Boston, MA USA; 9 Harvard Stem Cell Institute, Cambridge, MA USA; 10 Department of Psychiatry, Massachusetts General Hospital, Boston, MA USA; 11 Wyss Institute for Biologically Inspired Engineering, Harvard University, Boston, MA USA; 12 Department of Pathology, Brigham and Women’s Hospital, Harvard Medical School, Boston, MA USA; 13 Sackler School of Biomedical Sciences, Tufts University School of Medicine, Boston, MA USA; 14 Division of Rheumatology, Inflammation and Immunity, Brigham and Women’s Hospital, Boston, MA USA; 15 Medical Diagnostic Technology Evaluation, LLC, Carlisle, MA USA; 16 Center for Systems Biology, Massachusetts General Hospital, Boston, MA USA; 17 Department of Pathology, Massachusetts General Hospital, Boston, MA USA; 18 Wellman Center for Photomedicine, Massachusetts General Research Institute, Boston, MA USA; 19 Department of Dermatology, Massachusetts General Hospital, Boston, MA USA; 20 Evergrande Center for Immunologic Diseases, Brigham and Women’s Hospital, Boston, MA USA; 21 Cardiovascular Division, Department of Medicine, Brigham and Women’s Hospital, Boston, MA USA; 22 Functional Genomics Laboratory, Channing Division of Network Medicine, Brigham and Women’s Hospital, Boston, MA USA; 23 Center for Cancer Research, Massachusetts General Hospital, Harvard Medical School, Charlestown, MA USA; 24 Division of Nephrology and Endocrine Unit Department of Medicine, Massachusetts General Hospital, Boston, MA USA; 25 Cancer Center Protocol Office, Massachusetts General Hospital, Boston, MA USA; 26 Division of Preventative Medicine, Brigham and Women’s Hospital, Harvard Medical School, Boston, MA USA; 27 Radiology and pathology, Massachusetts General Hospital, Harvard Medical School, Boston, MA USA; 28 Brigham Research Institute, Brigham and Women’s Hospital, Boston, MA USA; 29 Immunology Program, Harvard Medical School, Boston, MA USA; 30 Cellular Immunotherapy Program, Cancer Center, Massachusetts General Hospital, Boston, MA USA; 31 Department of Radiation Oncology, Massachusetts General Hospital, Harvard Medical School, Boston, MA USA; 32 Folia Health, Inc., Cambridge, MA USA; 33 Vincent Center for Reproductive Biology, Department of Obstetrics and Gynecology, Massachusetts General Hospital, Boston, MA USA; 34 Department of Biology, Northeastern University, Boston, MA USA; 35 College of Science, Northeastern University, Boston, MA USA; 36 Mass General Brigham COVID Center for Innovation, Diagnostics Accelerator, Boston, MA USA

**Correction to: BMC Infect Dis 21, 580 (2021)**

**https://doi.org/10.1186/s12879-021-06257-7**

Following publication of the original article [1], the authors identified an error in the author’s name of Rakiesha Gay.

The incorrect name is:

- Rakeisha Gay

The correct name is:

- Rakiesha Gay

The author group has been updated above and the original article [1] has been corrected.
